# The Fee for a Denture Made on a Commercial Basis, Versus One Made on a Professional Basis

**Published:** 1915-06

**Authors:** Wilbur E. Bailey, Chas. P. Brown


### THE FEE FOR A DENTURE MADE ON A COMMERCIAL BASIS, VERSUS ONE MADE ON A PROFESSIONAL BASIS.

READ BEFORE THE STUDENTS' DENTAL SOCIETY OF THE UNIVERSITY OF MICHIGAN, MARCH 29, 1915.

BY WILBUR E. BAILEY AND CHAS. P. BROWN.

In considering this proposition, we shall first state the generally used arguments used to fix costs and profits in the business world. Any statement we may make as to proper fees for a professional dentist to charge, it should be understood, we are taking it for granted that the work is of the highest grade that the dentist is capable of doing, and that he has had adequate education and training for his work. The dentist should carefully consider what the denture has actually cost him in materials and time put into its construction. This would, by commercial methods, determine what recompense he has a right to expect for any particular variety of dentures. (The cost of reaching the patients should include all regular overhead charges, such as office rents, etc.) In order to arrive at any just cost of a dental service, we must necessarily resort to statistics, with all their faults. Variations in expense will occur, of course, in different localities, as well as in the kind of patients served and breadth of experience of the operator. If we are to arrive at anything like a true estimate of first costs, we should determine the various costs of the personal equipment of the dentist, which is as essential as to know the first cost of an automobile to the manufacturer. We can perhaps estimate the cost of a dental education at $3,000. If this was paid for on borrowed money, the interest will be an annual cost to be reckoned at five per cent. interest, it will make an item of $150. This includes only the cost of the three years spent in college and the time lost while taking the course. Interest on the office equipment of $1,000 at five per cent. with a depreciation of ten per cent., would add another $150. The depreciation of ten per cent. should be considered very small, for an entire new outfit, in almost every particular, will be needed in less than ten years (perhaps). Office expenses, including rent and materials used would vary with every locality, but $1,000 would be a fair average office expense. Every man is entitled to a (salary or) living income, varying as to his peculiar needs, such as the number of people dependent on him, and his own expenses. To start with $1,500 would be a fair average salary, but this would not allow for much to be laid away for the time when one can no longer practice, neither would it take into account accidents and losses by bad business methods or unfortunate investments. From these figures we see that at least we should collect each year $2,800. Bad debts and unreckoned charities will raise this to $3,000, which it will cost each year to run the office, even when doing a comparatively small business. (I would call attention to the fact that the average dentist practices only about 30 years with an income of $3,000.) The average student graduates at about 22 years and should by reasonable care, practice until he is 70 and should increase his practice each year until he is 50 years old at least. There are 300 working days in the year, although few men are able to stand so severe a nervous strain and give efficient service to their patients without a suitable vacation, which is a man's duty to himself and his family. Thus we can safely estimate on a liberal basis, ten dollars for each day's work, or $3,000 per year, as a probable income. Seeing patients and advising them without fees will consume at least two hours each day, leaving about four or five hours of actual working time for which one may charge, making a fee of $2.00 or $2.50 an hour necessary to meet the day's expenses. If the practice is entirely confined to prosthetic restorations we should know how much time is consumed in the actual construction of a denture in order that we may arrive at a just fee for such services. If it takes an entire day to make a full denture, a partial would take nearly as long, according to the type of case, we should have to charge $10.00 for the time put into the construction alone. If the case were simple, perhaps less time would be needed and if complicated or mistakes were made, even more time would be needed. Some persons work rapidly and some very carefully and slowly. To make a detailed study, we may make the following estimate:

| | |
|---|---|
| Impressions, pouring and preparing, models. | 1 hour |
| Taking bite and mounting | 1 hour |
| Setting up teeth | 1 hour |
| Investing and separating | ½ hour |
| Packing | ½ hour |
| Vulcanizing with time regulator | 2 hours |
| Cleaning and finishing | 1 hour |
| Fitting | 1 hour |
| Total, less vulcanizing | 6 hours |

It would be absolutely necessary for one to spend at least three hours of his own time on the case, but a trained office helper could save three hours work. On this basis the cost of the denture would be as follows:

| | |
|---|---|
| Dentist's time, at $2.50 per hours........ | $ 7.50 |
| Helper's time, at 20 cents per hour...... | .60 |
| Cost of teeth, vulcanite, etc.............. | 4.00 |
| Total, after allowing all profits....... | $12.10 |

From this estimate it will be seen that, a full vulcanite denture should not be offered for less than $12.00 if one expects to meet his obligations and realize a bookkeeper's salary. As a matter of fact, very few trained dentists will make dentures at such prices, as they can keep busy with more profitable work. Consequently, the mechanical dentist and advertisers, or the commercial laboratory makes our plates. Such dentists are willing to cut prices, make up their profits on other work, which is practically extending charity to their plate patients at the expense of patients requiring other than prosthetic services. We doubt if there are many patients who would not be willing to pay, when they are convinced, what a properly made denture is worth. We seen no reason why as professional men we can not charge what our services are worth to our patients, if they can afford it. There is no better service we can render our edentulous patients than a properly constructed substitute for his lost teeth. An old man needs nutrition as much as his younger friends and he can only have it by an adequate mastication of properly selected foods.

How can we make our patients realize the value of properly constructed dentures? We would first like to suggest that we discover the true value of a good denture to the patient. This is the important and perhaps the first question the dentist is required to answer satisfactorily. And it is worth while, if the dentist is competent to convince his patient, that the worth of the plate is more than the fee he pays for it. We can with most people make a strong and convincing argument from the esthetic side, the restoration of facial contour and artistic expression sometimes even improving on nature's handiwork. This can not fail to enlist their interest. If we can promise a proper facial contour and thus add to the outward appearance, we shall at least ingratiate ourselves to that extent. The artistic selection of appropriate teeth and their placement will greatly overcome the disfigurement one would necessarily experience without them. We can always make a strong appeal to the pride in one's personal appearance. The physiological effect produced by having a replacement of the lost function is a very important one, and is an appeal that can always be made to the intelligent and thoughtful people, the kind that have a keen sense of proportionate values. Mastication is the first step in the process of digesting the starchy foods at least. Bolting starchy foods is the crime of the ages. If we actually believe peoples' lives are lengthened by a proper mastication, artificial dentures to take the place of lost teeth is the only known resource when the natural teeth are lost. The advantages of well made dentures can hardly be overstated. In fact, dentists do not present this matter forcibly enough to their patients as a rule. The whole subject has been so discredited by commercial competition that it has almost lost interest to the true professional man. The time seems to be coming when the pendulum will swing back from so much crown and bridge work to the more healthy plate prosthesis. If we could realize that a properly constructed denture is often more sanitary and more justifiable than any so called bridge can be, we would surely strive to better this department of our service.

Too little thought is given by the average dentist to the selection of a proper material for cases where one material has decided preferences over another. The cost of plates has made the cheapest construction the prevailing method.

There are a number of different base plate materials, each of which has distinct advantages in certain places or conditions. There are also a number of combinations and methods of construction each of which has a distinct and valuable function in the art of prosthesis. We have the vulcanite, the combined aluminum and vulcanite, the cast metal, gold and vulcanite attachment, celluloid, continuous gum, etc. So that there is a wide range of selection of materials and methods of contruction from the inexpensive to the most expensive and likewise most artistic continuous gum plate. Good reasons for advising any one of these plates can be given. We fear the reason patients are not given this option of choice in many instances is not the implied cost so much as the dentist's incompetence. The competent professional dentist will recommend the kind of denture that the patient needs and should not offer compromises to satisfy the patient's ideas of price. Such advice and service must receive adequate compensations and usually where a patient is made intelligent on this matter, he will be more than glad to do his part. Another important question which is sure to arise, is, should the dentist give a definite price or fee to every patient for every kind and piece of work he may wish made? As a business detail, it is usually necessary to tell patients at least the approximate cost of a service that may be unusual. He may, if he thinks it necessary, also tell his patient he will use the best material and skill at his command and devote the proper amount of time required to provide the best piece of work he can produce. New patients ought to have all such information and such assurance as will enlist their co-operation in a desirable result. The values to be put into a prosthetic service should have just as good and adequate compensations as any other dental service of equal skill and value to the patient. If the values are given there rarely happens a disagreement on the basis of money compensation. One of our present day difficulties is lack of knowledge of differences as to values in artificial dentures. Most people see no difference between a skillful piece of workmanship and a showy and well polished denture of inferior quality in conception or adaptation. The public does not seem to know the difference between well made and adapted dentures and poor ones. They seem to think a plate is a plate whether it is in the mouth or on the sideboard. If a plate can be made that stays in place in the patient's mouth while eating, it is considered a success by the patient, although it may be faulty in materials, workmanship, adaptation and every possible sanitary standpoint. It should be one of the chief functions of a dentist to advise such patients as to the conditions under which they are tolerating such dentures. Now, if the dentist is to exercise the functions indicated in the discussion, is it reasonable to ask that he make such restorations at a price fixed by public opinion, formed from reading the advertisements of the quack dentist? Or can he in justice to himself and the traditions of his profession permit himself to slight in any particular a service which means so much for the well-being of his patient because he is compelled to put a price on his services in advance of rendering it? Isn't it high time for dentists to either quit the business of making artificial dentures, or, demand and get a fee rather than a price for all the services he renders of whatever character? With full knowledge of the conditions we shall have to meet in beginning a new practice in this matter, ought we not to spend the spare time that we may have in educating our patients to the idea of better prosthesis and in perfecting our skill in rendering sevices that will justify fees rather than prices?

